# Functional Characterization of the Cat and Dog Wild‐Type and Mutant MDR1 Carrier Proteins and Frequency of the *MDR1* Gene Mutation in 800 Cats From Germany

**DOI:** 10.1111/jvp.70041

**Published:** 2025-12-31

**Authors:** Lisa Siegl, Mies Bethäuser, Daniela Nürnberger, Stefan Oswald, Andreas Moritz, Melanie Hamann, Joachim Geyer

**Affiliations:** ^1^ Institute of Pharmacology and Toxicology, Faculty of Veterinary Medicine, Biomedical Research Center Seltersberg (BFS) Justus Liebig University of Giessen Giessen Germany; ^2^ Institute of Pharmacology and Toxicology, Rostock University Medical Center University of Rostock Rostock Germany; ^3^ Clinic of Small Animals—Internal Medicine, Faculty of Veterinary Medicine Justus Liebig University Giessen Giessen Germany

**Keywords:** digoxin, drug sensitivity, HEK293, ivermectin, MDCK, MDR1 (ABCB1), mutation, P‐glycoprotein, transport, transwell

## Abstract

The ATP‐binding cassette transporter MDR1 P‐glycoprotein (syn. ABCB1) is an efflux carrier at the cell membrane that regulates drug absorption, distribution, and elimination. At the blood–brain barrier, MDR1 restricts brain entry of potentially neurotoxic drugs, such as ivermectin. In dogs and cats, *MDR1* (syn. *ABCB1*) gene deletion mutations exist that have been associated with increased neurological toxicity after ivermectin treatment. The present study found an allelic frequency of 0.625% for the *MDR1* mutation in 800 cats from Germany. In addition, the canine and feline mutant and wild‐type MDR1 proteins were expressed in HEK293 and MDCKII cells, and transport experiments were performed with the fluorescent MDR1 probe substrate rhodamine 123. In both cell lines, significant MDR1‐mediated rhodamine 123 efflux was identified for the wild‐type MDR1 proteins, but not for the mutant MDR1 proteins, confirming a complete loss‐of‐function phenotype due to *MDR1* gene mutation. Competitive in vitro studies showed inhibition of both wild‐type MDR1 carriers with the reference MDR1 inhibitors verapamil (IC_50_ = 5–9 μM), PSC833 (IC_50_ = 1–2 μM), and tariquidar (IC_50_ = 0.1–0.2 μM), as well as with the antiparasitic drugs ivermectin (IC_50_ = 3–4 μM), eprinomectin (IC_50_ = 3–4 μM), moxidectin (IC_50_ = 8–21 μM), selamectin (IC_50_ = 10–22 μM), lotilaner (IC_50_ = 11–23 μM), and sarolaner (IC_50_ = 30–57 μM), clearly demonstrating multi‐drug interactions with the MDR1 carriers from both species.

## 1 Introduction

**FIGURE 1:**
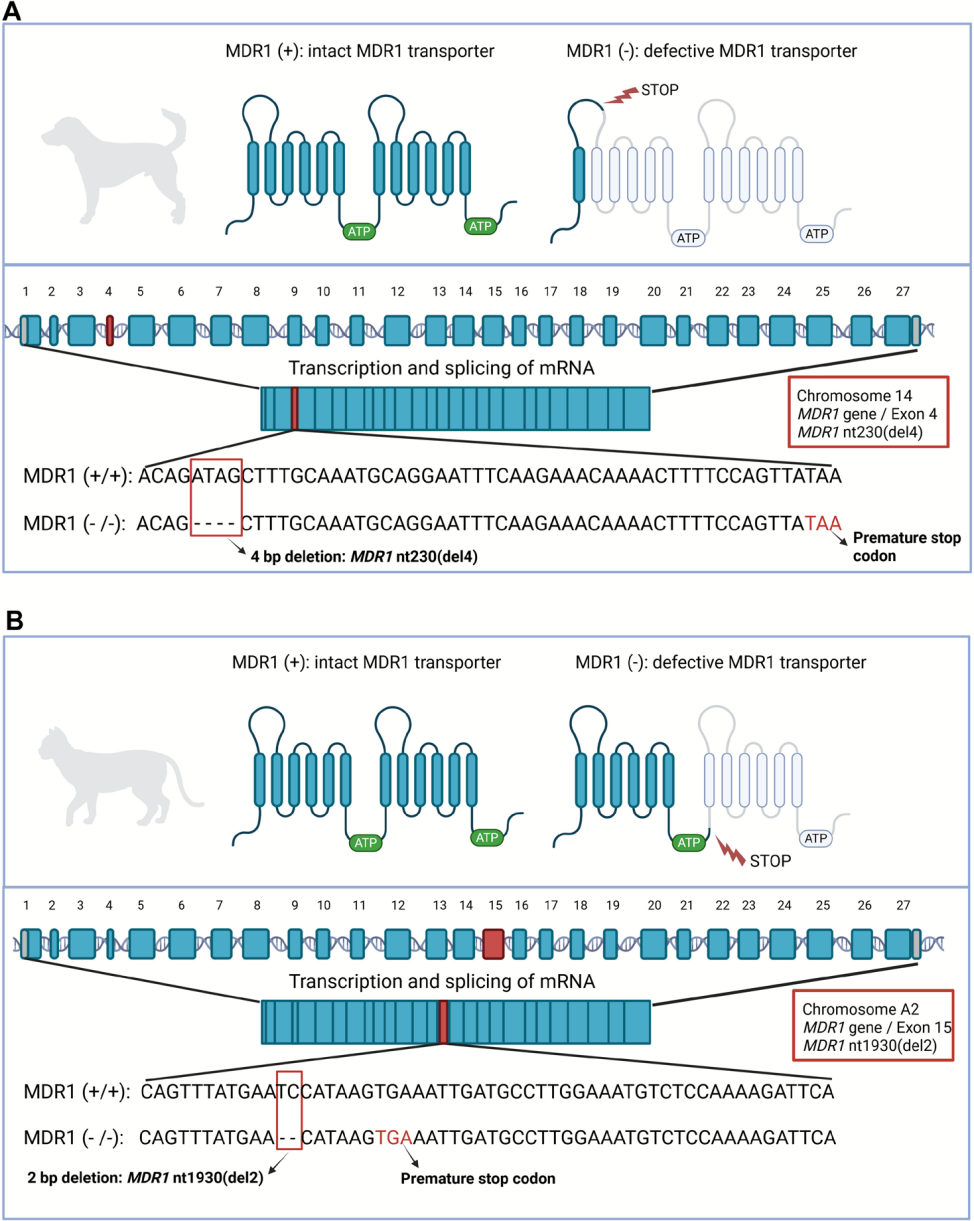
The wild‐type and mutant *MDR1* genes and MDR1 carrier proteins in dogs and cats. (A) In dogs, the nt230(del4) *MDR1* mutation occurs at nucleotide 230 of the open reading frame in exon 4 of the *MDR1* gene. The 4‐bp deletion results in a premature stop codon (TAA) that dramatically truncates the MDR1 carrier protein. (B) In cats, the nt1930(del2) *MDR1* mutation occurs at nucleotide 1930 of the open reading frame in exon 15 of the *MDR1* gene. The 2‐bp deletion results in a premature stop codon (TGA) and truncates the MDR1 carrier protein to half its normal size.

The multidrug resistance carrier MDR1 (syn. P‐glycoprotein, further referred to as MDR1 carrier) is a multidrug efflux carrier and phylogenetically belongs to the superfamily of ATP‐binding cassette (ABC) transporters (member ABCB1) (Dean and Annilo 2005). The MDR1 carrier protein is coded by the *ABCB1* gene (further referred to as *MDR1* gene). MDR1 transports many drugs with clinical relevance in veterinary medicine, including ivermectin, moxidectin, milbemycin oxime, selamectin, emodepside, cyclosporine A, butorphanol, loperamide, acepromazine, apomorphine, grapiprant, maropitant, vincristine, and doxorubicin (Geyer and Janko 2012; Mealey et al. 2017, 2023; Mealey and Burke 2025). The MDR1 carrier is highly expressed at the apical membrane of brain capillary endothelial cells and in the brush border membrane of enterocytes (Cordon‐Cardo et al. 1990; Ginn 1996). At these barriers, MDR1 restricts drug permeation and thereby limits oral drug bioavailability and brain penetration at the blood–brain barrier (Mealey 2004). In addition, the MDR1 carrier is actively involved in the hepatobiliary and renal drug excretion and overall plays a significant role for the pharmacokinetics of many drugs and toxins (Schinkel et al. 1997; Fromm 2000; Martinez et al. 2008). In veterinary medicine, the MDR1 carrier is of particular interest, as gene deletion mutations in the *MDR1* gene have been described for dogs (Mealey et al. 2001) and cats (Mealey and Burke 2015). In dogs, the *MDR1* nt230(del4) 4‐base pair (4‐bp) deletion in exon 4 of the *MDR1* gene (syn. MDR1‐1Δ or ABCB1‐1Δ) leads to an early stop codon already at amino acid position 91 of the 1281 amino acid spanning wild‐type MDR1 carrier protein (Mealey et al. 2001; Geyer, Döring, Godoy, Moritz, et al. 2005) (Figure 1A). In cats, the *MDR1* nt1930(del2) 2‐bp deletion in exon 15 of the *MDR1* gene (syn. ABCB1 1930_1931del TC) truncates the wild‐type cat MDR1 protein of 1279 amino acids to its first half representing amino acids 1–643 (Mealey and Burke 2015; Nürnberger et al. 2022) (Figure 1B). Occurrence of this mutation has intensively been investigated in the dog population in many countries and revealed the highest prevalence for the homozygous mutation in Collie dogs. In addition, the nt230(del4) *MDR1* mutation has been detected in the following dog breeds: Australian Shepherd, Miniature Australian Shepherd, Black Mouth Cur, Border Collie, Boxer, Carolina dog, Chinook, English Shepherd, German Shepherd, Golden Retriever, Longhaired Whippet, McNab, Old English Sheepdog, Shetland Sheepdog, Siberian Husky, Silken Windhound, Wäller, and White Shepherd (Neff et al. 2004; Geyer, Döring, Godoy, Leidolf, et al. 2005; Geyer, Döring, et al. 2007; Mealey and Meurs 2008; Gramer et al. 2011; Mealey et al. 2023). In contrast, prevalence of the nt1930(del2) *MDR1* mutation in cats is less well characterized. In some studies, this *MDR1* mutation was identified in cats sensitive to drugs like ivermectin and eprinomectin (Mealey and Burke 2015; Mealey et al. 2021, 2024, 2025; Nürnberger et al. 2022). In addition, a recent study analyzed 1006 banked feline DNA samples from the United States and found the heterozygous *MDR1* mutation in 47 cats and an overall allele frequency of the *MDR1* nt1930(del2) mutant allele of 4.7% (Mealey et al. 2021). Affected cats were purebred Ragdoll, Russian Blue, and Siamese cats. In another large DNA genotyping study in 11,036 cats from the United States and Finland, 123 cats revealed a heterozygous and 3 cats revealed a homozygous *MDR1* mutation, resulting in an overall allele frequency of the *MDR1* nt1930(del2) mutant allele of 0.6% (Anderson et al. 2022). In this study, the *MDR1* mutation was detected in purebred Siamese, Balinese, Main Coon, Maine Coon Polydactyl, and Turkish Angora cats. Dogs and cats with homozygous *MDR1* gene deletion mutation are highly drug sensitive (Mealey 2004). In dogs, this is most severe when antiparasitic macrocyclic lactone drugs are used off label, such as ivermectin at 0.2 mg/kg (oral application) or doramectin at 0.6 mg/kg (subcutaneous injection). Both treatments often result in fatal cases of neurological toxicity (Mealey et al. 2001; Hopper et al. 2008; Geyer and Janko 2012). In cats, severe neurological toxicity has also been described for approved eprinomectin‐containing formulations (Mealey et al. 2021, 2024, 2025), clearly underlines the clinical importance for *MDR1* genotyping and genotype‐based drug selection and dosing in canine and feline medicine (Geyer and Janko 2012; Mealey et al. 2023).

In the present study, we established HEK293 and MDCKII cell lines stably transfected with the wild‐type and mutant MDR1 constructs from dogs and cats. While the cell lines expressing the wild‐type MDR1 proteins were used for drug interaction studies with antiparasitic drugs of the macrocyclic lactone (ivermectin, eprinomectin, moxidectin, selamectin, and milbemycin oxime) and isoxazoline (lotilaner, sarolaner, fluralaner, afoxolaner) classes of antiparasitic drugs, the mutant MDR1 proteins were used to verify the loss‐of‐function phenotype. In addition, the frequency of the *MDR1* gene mutation was analyzed in 800 cat patients of a university veterinary clinic in Germany.

## 2 Material and Methods

### 2.1 Materials

All materials if not stated otherwise were obtained from Sigma‐Aldrich (St. Louis, MO, USA). Rhodamine 123 (Rh123) was obtained from Abcam (Cambridge, UK). Radio‐labeled [^3^H]digoxin (specific activity 40 Ci/mmol) was obtained from Perkin Elmer (Shelton, CT, USA). Antiparasitic drugs of the macrocyclic lactone and isoxazoline classes except for eprinomectin were obtained from HPC Standards (Cunnersdorf, Germany). Eprinomectin was obtained from Selleckchem (Houston, TX, USA). The MDR1 control inhibitor verapamil was obtained from Biotrend (Cologne, Germany) and PSC833 was from Cayman Chemicals (Ann Arbor, Michigan, USA).

### 2.2 Cell Culture

Human embryonic kidney Flp‐In T‐REX cells (HEK293‐FlpIn) (Thermo Fisher Scientific, Carlsbad, CA, USA) and transfected MDR1‐HEK293 cells were maintained in Dulbecco's modified Eagle medium with F12 nutrient mixture (DMEM/F12, Gibco, Carlsbad, CA, USA) supplemented with 10% fetal calf serum (FCS, Gibco), 2 mM l‐glutamine (Gibco), 100 U/mL penicillin (Gibco), and 100 μg/mL streptomycin (Gibco). The Madin‐Darby canine kidney (MDCKII) MDR1 knockout cell line (hereafter referred to as MDCKII^MDR1ko^) has been engineered by CRISPR/Cas9 mutation resulting in a complete knockout of the *MDR1* gene and was purchased from Sigma‐Aldrich. Non‐genetically modified MDCKII cells were also from Sigma‐Aldrich. All MDCKII cell lines were cultured in MEM medium (Gibco), supplemented with 10% FCS (Gibco), 2 mM l‐glutamine (Gibco), 100 U/mL penicillin (Gibco), and 100 μg/mL streptomycin (Gibco). All cell lines were maintained at 37°C, 5% CO_2_, and 95% humidity.

### 2.3 Stably Transfected Dog and Cat MDR1‐HEK293 Cell Lines

The canine and feline MDR1 cDNA clones were obtained in the context of diagnostic full‐length MDR1 sequencing as reported before (Elmshäuser et al. 2015; Gramer et al. 2015; Nürnberger et al. 2021, 2022). The wild‐type constructs as well as the dog nt230(del4) and cat nt1930(del2) mutant constructs were used for stable transfection of HEK293‐FlpIn as reported before (Geyer, Döring, et al. 2007; Elmshäuser et al. 2015). Briefly, the pcDNA5‐based expression vectors were transfected into the HEK293‐FlpIn using Lipofectamine 2000 transfection reagent (Thermo Fisher Scientific). The transfection procedure was performed in six‐well plates at 80%–90% confluence. Transfected cell clones were selected with 100 μg/mL hygromycin B (Carl Roth, Karlsruhe, Germany) treatment, and single hygromycin B resistant cell clones were further cultivated and checked for transgene expression by quantitative mRNA expression analysis. In the generated dog and cat MDR1‐HEK293 cell lines, the expression of the transgene is under control of a tetracycline‐regulated promoter. Gene expression from these HEK293 cells was induced with 1 μg/mL tetracycline (Carl Roth) treatment.

### 2.4 Stably Transfected Dog and Cat MDR1‐MDCKII Cells Lines Based on FRT‐Competent MDCKII^MDR1ko^ Cells

Commercially obtained MDR1‐deficient MDCKII^MDR1ko^ cells were in a first step stably transfected with the Flp recombinase target (FRT) site using the Flp‐In system according to the standard protocol (Thermo Fisher Scientific). Briefly, cells were seeded in 24‐well plates and were stably transfected with the linearized pFRT/lacZeo vector (Thermo Fisher Scientific) using Lipofectamine 2000 (Thermo Fisher Scientific). Then, cells were selected for transgene insertion with 400 μg/mL zeocin (Thermo Fisher Scientific) treatment. Single clones were isolated, and their beta‐galactosidase activity was measured using the beta‐galactosidase enzyme assay system from Promega (Madison, WI, USA). The cell clones with the highest beta‐galactosidase activity were selected and used for generation of stably transfected dog and cat MDR1‐MDCKII cell lines. For stable transfection of the MDCKII‐FlpIn cells, the same procedure was used as described above for the HEK293‐FlpIn cells.

### 2.5 *MDR1* mRNA Expression Analysis in Stably Transfected HEK293 and MDCKII Cells

Stably MDR1‐transfected HEK293 and MDCKII cells were harvested, and total RNA was extracted by the Quick‐RNA kit (Zymo Research, Irvine, CA, USA) according to the manufacturer's protocol. Complementary cDNA was synthesized from 1 μg total RNA by using 7 μL of RT‐mix SuperScript III reverse transcriptase (Thermo Fisher Scientific). The PCR amplification was performed in a Peqlab peqSTAR thermal cycler (VWR, Radnor, PA, USA) with the following conditions: 95°C for 10 min, followed by 40 cycles of 15 s at 95°C and 60 s at 60°C. The TaqMan gene expression assays (Thermo Fisher Scientific) used for quantitative expression analysis are listed in Table 1. Relative gene expression analysis was performed with QuantStudio 1 (Thermo Fisher Scientific). Data were expressed as fold changes using the 2^−ΔΔCt^ or ΔCt methods as reported before (Geyer, Döring, et al. 2007).

**TABLE 1:** TaqMan probes used for *MDR1* mRNA expression analysis in stably transfected HEK293 and MDCKII^MDR1ko^ cells by real‐time PCR.

TaqMan gene expression assay	Gene	Based on GenBank accession number	Specific for
hs99999905_m1	*hGAPDH*	NM_001289746.1	Human
cf02659079_m1	*cfB2M*	XM_003640047.2	Dog
cf02693309_m1	*cfMDR1 (cfABCB1)*	NM_001003215.2	Dog and human
cf02622140_m1	*cfMDR1 (cfABCB1)*	NM_001003215.2	Dog
MDR1CatbpExon15	*fcMDR1 (fcABCB1)*	NM_001171064.2	Cat

Abbreviations: B2M, beta‐2‐microglobulin; cf., *canis familiaris* ; fc, *felis catus* ; GAPDH, glycerinaldehyde‐3‐phosphate‐dehydrogenase; h, human.

### 2.6 MDR1 Protein Expression Analysis in Stably Transfected HEK293 Cells

MDR1 protein expression in the stably transfected MDR1‐HEK293 cells was analyzed by mass spectrometry (MS)‐based targeted proteomics. Briefly, cell pellets were lysed and homogenized using a glass Dounce homogenizer. Total protein determination was performed using the BCA protein assay kit (Millipore, Billerica, MA, USA). Sample cleanup was performed according to a filter aided sample preparation (FASP) protocol. Briefly, 10 K filter units (Sartorius, Goettingen, Germany) were first centrifuged with 1% formic acid for 15 min. A protein amount of 100 μg was adjusted to 200 μL with UT buffer (8 M urea, 2 M thiourea in aqua destillata) and then added to the filter units, followed by a centrifugation step at 14,000×*g* for 30 min at room temperature (RT). All subsequent centrifugation steps were performed at 14,000×*g* at RT. After the addition of 200 μL urea buffer (8 M urea, 100 mM Tris in aqua destillata, pH 8.5) and 30 min of centrifugation, 100 μL DTT buffer (8 mM dithiothreitol in urea buffer) were added, and the probe was incubated at 56°C for 15 min, followed by 25 min of centrifugation. After the addition of 100 μL urea buffer and 25 min of centrifugation, 100 μL IAA buffer (50 mM iodoacetamide in urea buffer) were added, followed by incubation at RT in the dark for 20 min and 25 min of centrifugation. The centrifugation and incubation steps were repeated after the addition of urea buffer and DTT buffer. After the addition of 100 μL ABC buffer (65 mM ammonium bicarbonate in aqua destillata), the filter units were washed twice with 65 mM ammonium bicarbonate. Proteins were digested with trypsin at a protein‐to‐trypsin ratio of 1:100 at 37°C for 16 h. Clean collection tubes were used for peptide elution by centrifugation at 14,000×*g* for 10 min after the addition of 10 μL of 10% formic acid. Then, 40 μL of ABC buffer were added, followed by 10 min of incubation at 14,000×*g*. Eluted peptides were stored at −80°C until liquid chromatography–mass spectrometry (LC–MS/MS) analysis. All sample preparation steps were performed using protein LoBind tubes (Eppendorf, Hamburg, Germany). The following peptides were used for quantification of the human, canine, and feline MDR1 proteins: AGAVAEEVLAAIR, IATEAIENFR, and FYDPLAGK. LC–MS/MS proteomic analysis was performed on the Agilent 1290 HPLC system coupled to the Sciex QTRAP 7500 triple quadrupole mass spectrometer as described before (Groer et al. 2013). The final protein abundance data (in picomoles per milligram of protein) were calculated by means of normalization to the total protein content of the isolated membrane fraction.

### 2.7 MDR1 Protein Expression Analysis by Western Blotting

Cells were cultured in six‐well plates, then harvested and lysed in RIPA buffer (150 mM NaCl, 50 mM Tris, 1% NP‐40, 0.5% sodium deoxycholate, 0.1% SDS, pH 7.4), supplemented with protease inhibitor (Thermo Fisher Scientific). The lysates were normalized for protein content by BCA assay according to the manufacturer's protocol. The samples with 75 μg protein per lane were separated by sodium dodecyl‐polyacrylamide gel electrophoresis (SDS‐PAGE) on 8% gels and then transferred to a polyvinylidene fluoride membrane (PVDF) (Carl Roth). The membrane was blocked with 5% milk solution for 1 h at RT, incubated with primary antibodies overnight at 4°C and then washed four times with TBS‐T buffer (137 mM NaCl, 10 mM Tris base, 0.05% Tween‐20, pH 8.0). After incubation with the secondary horseradish peroxidase‐IgG antibodies, the blots were imaged in chemiluminescent solution containing Super Signal West Pico Plus Chemiluminescent Substrate (Thermo Fisher Scientific) on a Chemidoc imaging system (Bio‐Rad, Hercules, CA, USA). The following antibodies were used: mouse anti‐MDR1 primary antibody C219 (dilution 1:1000, Cat# 517310, Calbiochem, Billerica, MA, USA), mouse anti‐beta‐actin primary antibody (1:5000 dilution, Cat# A5441, Sigma‐Aldrich) and horseradish peroxidase‐conjugated rabbit anti‐mouse secondary antibody (1:5000 dilution, Cat# A9044, Sigma‐Aldrich).

### 2.8 Transport Experiments in HEK293 Cells With Rhodamine 123 (Rh123)

Transport experiments in the stably transfected MDR1‐HEK293 cells were performed with the fluorescent MDR1 substrate rhodamine 123 (Rh123). Cells were seeded onto polylysine‐coated 24‐well plates. Gene expression was induced with 1 μg/mL tetracycline treatment. Cells were grown to confluence over 72 h at 37°C. Then, cells were washed with 37°C tempered phosphate buffered saline (PBS, containing 137 mM NaCl, 2.7 mM KCl, 1.5 mM KH_2_PO_4_, 7.3 mM Na_2_HPO_4_, pH 7.4). Subsequently, PBS was replaced with 250 μL of transport buffer (DMEM without phenol red) with or without the MDR1 inhibitor tariquidar (TQD) at a final concentration of 1 μM. After 30 min pre‐incubation, transport experiments were started by replacing the medium with transport buffer additionally containing 5 μM Rh123. The Rh123 uptake was stopped after 90 min by two‐times washing with ice‐cold PBS. Subsequently, the PBS was again replaced with 250 μL of DMEM as the transport buffer, with or without TQD, and the efflux phase lasted over 60 min at 37°C. Then, experiments were stopped by two‐times washing with ice‐cold PBS. Finally, cells were lysed with 500 μL of 1 N NaOH with 0.1% SDS. Cell‐associated fluorescence of Rh123 was quantified by GloMax microplate reader (Promega). Fluorescence was detected at 490 nm excitation and 510–570 nm emission. The protein content was determined according to Lowry using aliquots of the lysed cell with bovine serum albumin as standard (Lowry et al. 1951). The absolute fluorescence units per well were normalized to the protein amount and expressed as relative fluorescence units (RFU)/μg protein.

### 2.9 Inhibitor Screening and Determination of Half‐Maximal Inhibitory Concentrations (IC_50_ )

Transport inhibition experiments were performed with 5 μM Rh123 as the probe substrate in stably transfected MDR1‐HEK293 cells. Cells were seeded onto polylysine‐coated 96‐well plates and gene expression was induced with tetracycline treatment at 1 μg/mL. Cells were grown to confluence over 72 h at 37°C. Then, cells were washed with 37°C tempered PBS and preincubated for 30 min at RT with 80 μL of phenol red‐free DMEM as the transport buffer containing the respective inhibitor or solvent alone. Transport experiments were started by adding 20 μL of transport buffer containing 25 μM of Rh123 (final substrate concentration: 5 μM). The Rh123 uptake was stopped after 90 min by two‐times washing with ice‐cold PBS. Subsequently, the PBS was replaced with 80 μL of transport buffer again containing the respective inhibitor or solvent alone, and cells were further incubated for 60 min at 37°C for the efflux phase. Experiments were stopped by two‐times washing with ice‐cold PBS. Cell‐associated fluorescence of Rh123 was quantified by GloMax microplate reader (Promega). If not otherwise indicated, the inhibitory concentrations were as follows: 0.1, 0.3, 1, 3, 10, 30 and 100 μM. TQD was used as the MDR1 control inhibitor at 1 μM inhibitor concentration.

### 2.10 Transwell Transport Experiments in MDCKII Cells With Rh123 and [ ^3^H]Digoxin

For transwell transport experiments in the MDCKII cell lines, 3 × 10^5^ cells were seeded per 12‐mm/0.4 μm transwell membrane inserts (Sarstedt, Numbrecht, Germany). One day after seeding, the medium was changed. The transport experiments were performed 72 h after seeding and after reaching 100% confluence. Measurement of the transepithelial electrical resistance (TEER) was conducted on each well with the Millicell ERS‐2 electrical resistance system (Millipore) to confirm the integrity of the monolayer. Only cell monolayers that reached > 150 Ω cm^2^ were used for the transport experiments. As an additional test on monolayer integrity, permeability tests were performed with Lucifer Yellow (Thermo Fisher Scientific). For an intact cell monolayer, the Lucifer Yellow flux was at < 2%. Transport experiments were performed in both apical‐to‐basolateral (AB) and basolateral‐to‐apical (BA) directions. Before the assay was started, filter inserts were washed once with PBS to remove residual culture medium. Then, for AB transport experiments, 1.5 mL transport buffer (phenol red‐free DMEM) was added into the well, and 400 μL transport buffer was applied into the insert. After pre‐incubation of 30 min, transport experiments were started by adding 100 μL transport buffer containing 25 μM Rh123 (final concentration: 5 μM) into the insert. For BA transport experiments, 1.2 mL transport buffer was added into the well and 500 μL transport buffer was applied into the insert. After the 30 min pre‐incubation phase, transport experiments were started by adding 300 μL transport buffer with 25 μM Rh123 (final concentration: 5 μM) into the well. Samples were taken at separate times from the respective opposite compartments, that is, 150 μL from the well for the AB measurements and 50 μL from the insert for the BA measurements. The removed volumes were replaced with fresh transport buffer. The cells were incubated at 37°C and 5% CO_2_ in transport buffer during the entire experiment. The transwell transport experiments were performed in the presence and absence of the MDR1 inhibitor TQD. The inhibitor concentration of 1 μM TQD was maintained in both compartments throughout the experiment. All measurements were done in triplicates. Fluorescence of Rh123 was detected with the GloMax plate reader. Transport experiments with radio‐labeled [^3^H]digoxin followed the same procedure. In this case, the final substrate concentration was 1 μM and the transport buffer was supplemented with 1% bovine serum albumin (BSA). The amount of [^3^H]digoxin in the samples was quantified by Tri‐Carb 2910TR Liquid Scintillation Analyzer (Perkin Elmer).

For all transwell experiments with Rh123 and [^3^H]digoxin as the substrates, apparent permeability coefficients (*P*_app_, cm/s) and the efflux ratios (ER) were calculated as follows:

$$P_{\mathrm{app}}=\left(V/A\times C_{0}\right)\times \left(dQ/dt\right)$$

where “*V*” is the volume of the receiver chamber (1.5 or 0.5 mL), “*A*” is the surface area of the transwell inserts (being 1.1 cm^2^), “*C*_0_” is the initial concentration of the compound, and “d*Q*/d*t*” is the transport rate.

From the *P*_app_ values of the AB and BA transport experiments, the ER was calculated as follows:

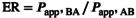




### 2.11 Genotyping of the nt1930(del2) *MDR1* Mutation in Cat Patients From a University Clinic

To determine the frequency of the nt1930(del2) *MDR1* mutation in cat patients in a university animal clinic in Germany, 800 surplus diagnostic blood samples from cats were collected from the Clinic for Small Animals of the Justus Liebig University Giessen. The DNA sampling for this study was reviewed and approved by the local animal welfare authority (Regierungspräsidium Giessen; registration number: 19 c 20 15 h 02 Gi 18/11 kTV 10/2021). Genomic DNA was extracted from 200 μL of EDTA‐preserved blood samples using the NucleoSpin Blood QuickPure Kit (Macherey‐Nagel, Düren, Germany). *MDR1* genotyping for the cat nt1930(del2) mutation was performed as previously reported (Nürnberger et al. 2022) and this assay is based on the previously published method to detect the nt230(del4) *MDR1* mutation in dogs (Klintzsch et al. 2010). Briefly, a total of 4.5 μL of genomic DNA was mixed with 5 μL of Genotyping Master Mix (2X) (Thermo Fisher Scientific) and 0.5 μL of TaqMan Assay Mix (20× working stock solution) (Thermo Fisher Scientific). Real‐time PCR was performed in a 96‐well reaction plate (Thermo Fisher Scientific) on a StepOnePlus Real‐Time PCR System (Thermo Fisher Scientific) under the following conditions: a pre‐PCR plate read at 60°C for 30 s, followed by a 95°C holding stage for 10 min, then 40 cycles of 95°C for 15 s and 60°C for 1 min, and finally a 60°C post‐PCR plate read for 30 s.

### 2.12 Statistics

All graphs and diagrams were generated with GraphPad Prism 6 (GraphPad Software Inc., San Diego, CA, USA). The IC_50_ values were determined by nonlinear regression analysis using the equation log (inhibitor) versus response—variable slope settings. Statistical analyses were performed using GraphPad Prism 6. As detailed in the figure legends, either Student's *t*‐test or two‐way ANOVA was applied, as appropriate. A *p*‐value < 0.05 was considered statistically significant.

## 3 Results

### 3.1 Establishment of Cell Lines for Functional MDR1 Analysis

The primary objective of this study was to establish cell lines suitable for analyzing drug interactions and drug transport mediated by the canine and feline MDR1 drug efflux transporters. Additionally, we aimed to determine whether the truncated MDR1 proteins resulting from the *MDR1* nt230(del4) gene mutation in dogs and the *MDR1* nt1930(del2) gene mutation in cats are still expressed in cell culture and whether they retain any residual transport activity. While functional activity of the severely truncated canine MDR1 protein appears to be completely abolished, it remains under discussion whether the mutated “half‐transporter” in MDR1‐mutant cats could form artificial homodimers with residual transport function.

### 3.2 Characterization of Stably Transfected HEK293 Cell Lines

**FIGURE 2:**
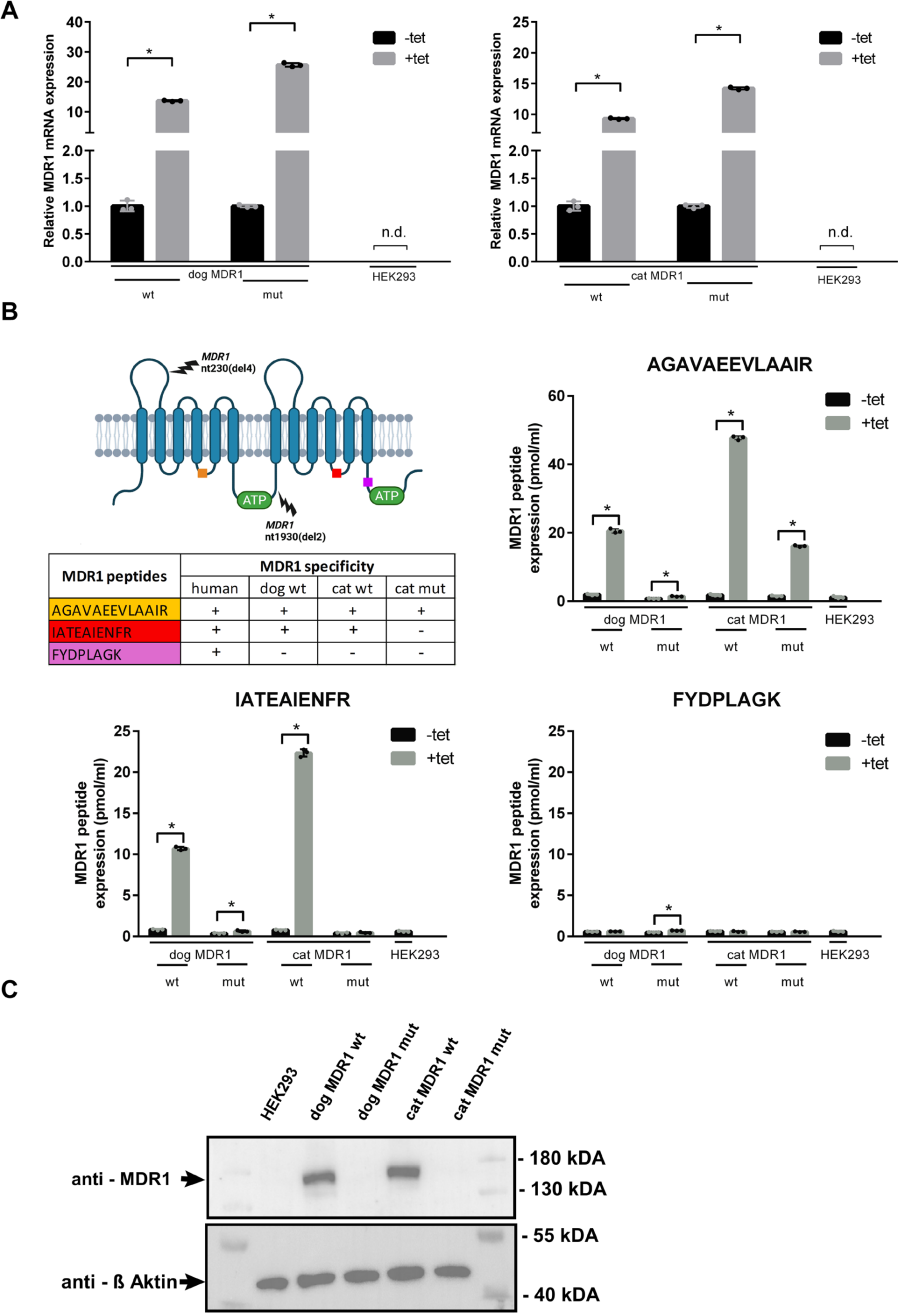
Wild‐type and mutant *MDR1* mRNA and MDR1 carrier protein expression in stably transfected HEK293 cells. (A) *MDR1* mRNA expression of the wild‐type (wt) and mutant (mut) *MDR1* transcripts of dog and cat were quantified by real‐time PCR from stably MDR1‐transfected HEK293 cells. Cells were treated with tetracycline (+tet) to induce *MDR1* mRNA expression or were untreated for control (−tet). The species‐specific TaqMan probes used for quantitative PCR amplification are listed in Table 1. Expression data were analyzed with the 2^−ΔΔCt^ method with untreated cells (−tet) as control set as calibrator. Data represent RQmin/RQmax from triplicate determinations. *Significantly different with *p* < 0.05 according to Student's *t*‐test; n.d., not detected. (B) Expression of the wild‐type and mutant MDR1 carrier proteins of dog and cat in the stably transfected HEK293 cells was analyzed by LC–MS/MS based targeted proteomics using the indicated reference peptides for quantification. The inset table provides an overview of the species‐specific occurrence of these reference peptides, and the transmembrane model indicates their positions in the full‐length MDR1 carrier protein. Proteomics analysis was performed in HEK293 cells stably transfected with the wild‐type or mutant MDR1 constructs from dog and cat as well as in untreated non‐transfected HEK293 control cells. The transfected cell lines were treated with tetracycline (+tet) to induce MDR1 carrier protein expression or were untreated for control (−tet). Data represent means ± SD of triplicate determinations. *Significantly different with *p* < 0.05 according to Student's *t*‐test. (C) Detection of the wild‐type MDR1 carrier proteins of dog and cat by Western blot analysis of stably transfected HEK293 cells with the C219 antibody.

**FIGURE 3:**
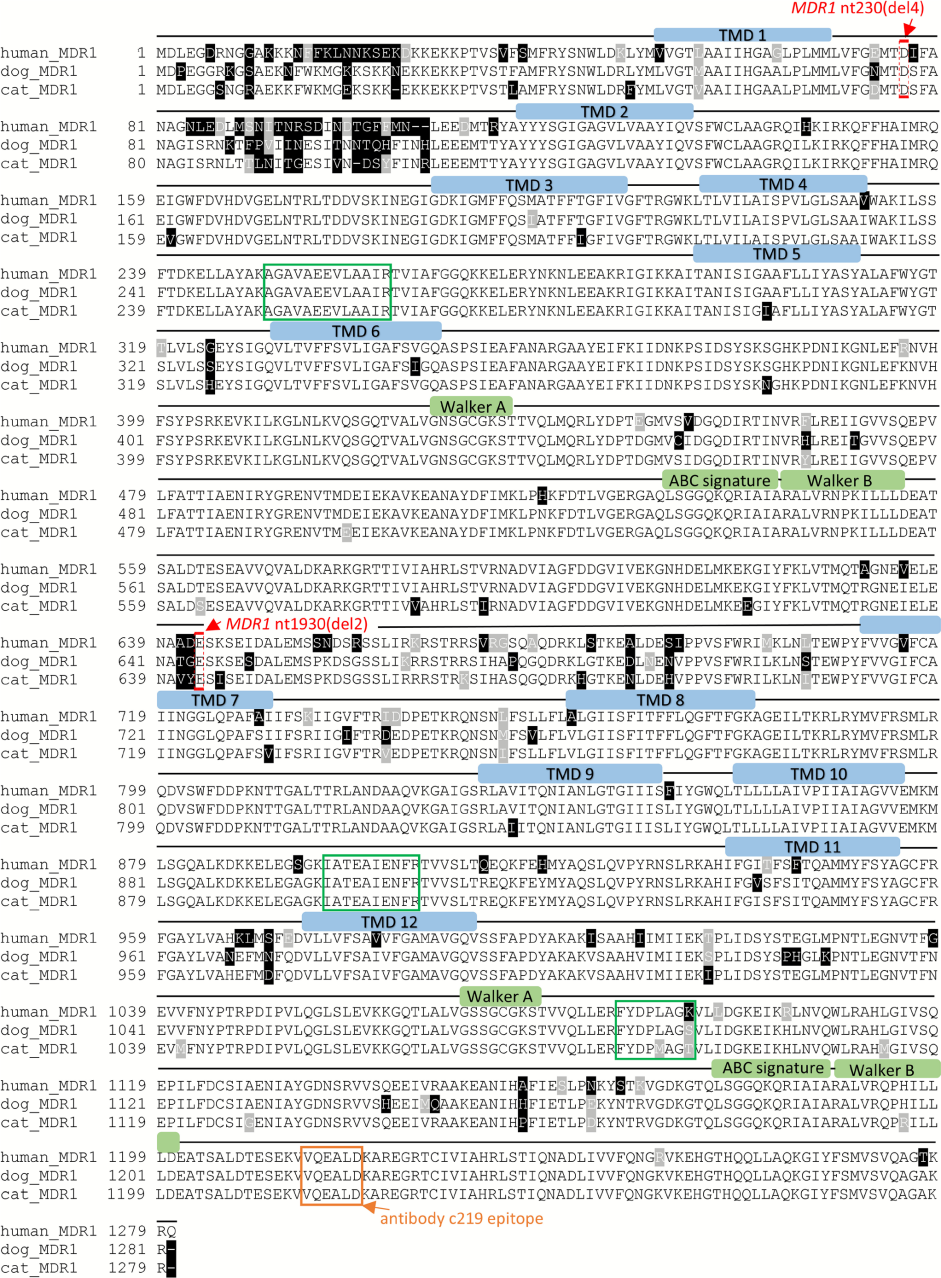
Sequence alignments of human, cat, and dog MDR1 carrier proteins. The amino acid sequences of human MDR1 (GenBank Accession No. NP_000918.2), dog MDR1 (GenBank Accession No. NP_001003215.2) and cat MDR1 (GenBank Accession No. NP_001164535.1) were aligned using the ClustalW algorithm in the DNASTAR 16.0 software. Visualization was performed using BOXSHADE. Localization of the *MDR1* nt230(del4) and *MDR1* nt1930(del2) mutations (red box) are indicated at the protein level. In addition, the MDR1 peptides used for mass spectrometry‐based targeted proteomics are indicated with green boxes and the epitope of the C219 MDR1 primary antibody is indicated with an orange box. Transmembrane domains of the MDR1 carrier protein are labeled with blue bars and the conserved motifs Walker A, Walker B, and C motif (green bars) are highlighted.

As a first step, HEK293 cell lines stably expressing wild‐type and mutant *MDR1* transcripts from dogs and cats were established. In these cell lines, expression of the respective *MDR1* transcripts is inducible by tetracycline. To quantify species‐specific *MDR1* mRNA expression above the endogenous background *MDR1* mRNA from the human‐derived HEK293 cells, we used gene expression assays specific to the canine (assay cf02622140_m1) and feline (MDR1CatbpExon15) *MDR1* transcripts (see Table 1). As shown in Figure 2A, these transcripts were not detectable in parental HEK293 cells. In contrast, all stably transfected cell lines exhibited a significant upregulation of species‐specific *MDR1* mRNA transcripts following tetracycline (+tet) induction. To determine whether MDR1 carrier proteins were also expressed from these transcripts, we conducted targeted proteomic analyses using three MDR1‐specific peptides. The peptide AGAVAEEVLAAIR was used to detect MDR1 proteins from human, dog (wild‐type MDR1), and cat (wild‐type and mutant MDR1). The second peptide, IATEAIENFR, specifically detected MDR1 proteins from human, canine (wild‐type MDR1), and feline (wild‐type MDR1) origin (Figure 2B). The third peptide, FYDPLAGK, was specific to human MDR1 and served as a control for detecting the endogenous MDR1 background expression in all HEK293 cell lines. As illustrated in Figure 2B, expression of the wild‐type MDR1 carrier proteins of dog and cat was clearly confirmed, and importantly, expression of the truncated mutant cat MDR1 protein was also detected. All cell lines showed low‐level background expression of the human MDR1 protein. Notably, only in HEK293 cells expressing the mutant canine MDR1 protein did tetracycline induction result in a slight but significant increase in endogenous human MDR1 protein expression. Furthermore, expression of the recombinant wild‐type MDR1 carrier proteins of dog and cat was confirmed by Western blot analysis using the C219 antibody, which recognizes a C‐terminal epitope present in the human, canine, and feline MDR1 proteins (see Figure 3). As expected, this antibody was able to detect the wild‐type MDR1 proteins, but not the mutant MDR1 proteins. The endogenous human MDR1 protein expression from HEK293 cells was below the detection threshold of Western blot analysis. In contrast, both wild‐type MDR1 carrier proteins of dog and cat were clearly detected (Figure 2C).

### 3.3 Efflux Function of Wild‐Type Canine and Feline MDR1 Carriers in Stably Transfected HEK293 Cells

**FIGURE 4:**
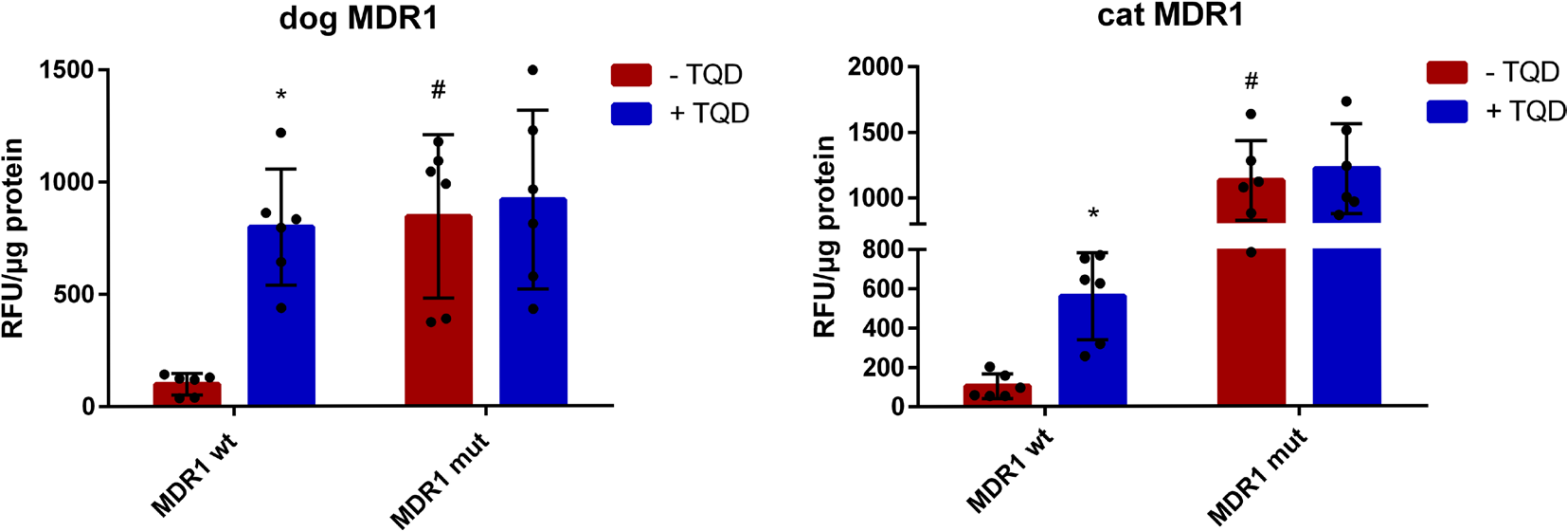
Transport experiments with Rh123 in HEK293 cells stably transfected with the wild‐type and mutant MDR1 constructs of dog and cat. All transport experiments were performed with 5 μM Rh123 in tetracycline‐treated HEK293 cells expressing the canine MDR1 wild‐type (dog MDR1 wt) or nt230(del4) MDR1 mutant (dog MDR1 mut) constructs, as well as the feline MDR1 wild‐type (cat MDR1 wt) or nt1930(del2) mutant (cat MDR1 mut) constructs, respectively. The Rh123 efflux was determined in the presence (blue bars) and absence (red bars) of the MDR1 inhibitor tariquidar (TQD) using a fluorescent microplate reader. Low fluorescence measured as relative fluorescent units (RFU) indicates high MDR1‐mediated efflux rates. Data represents SD from three independent experiments, each with duplicate determinations. *Significantly higher Rh123 accumulation in the presence of TQD and ^#^significantly higher Rh123 accumulation for the MDR1 mutant compared to the wild type according to two‐way ANOVA with *p* < 0.05.

The stably transfected HEK293 cell lines were subsequently used to assess efflux activity using the prototypical MDR1 substrate rhodamine 123 (Rh123). In this assay, active MDR1‐mediated efflux prevents intracellular accumulation of Rh123, resulting in low cellular fluorescence levels. Conversely, high intracellular fluorescence indicates impaired or absent efflux function. The MDR1‐specific inhibitor tariquidar (TQD) was used as a control to confirm MDR1‐specific transport activity. In cells expressing functional MDR1 carrier proteins, TQD treatment is expected to inhibit efflux, thereby increasing Rh123 accumulation and cellular fluorescence. As shown in Figure 4, the HEK293 cell line expressing the mutant canine MDR1 protein exhibited high intracellular Rh123 fluorescence, consistent with a complete loss of efflux function. In contrast, the cell line expressing the wild‐type canine MDR1 carrier proteins showed significantly reduced fluorescence levels, indicating active efflux of Rh123. This activity was fully abolished upon treatment with TQD, confirming the functional specificity of the transporter. Similarly, the wild‐type feline MDR1 carrier mediated a significant, TQD‐sensitive reduction in Rh123 accumulation, demonstrating robust efflux activity. In contrast, the truncated mutant feline MDR1 protein displayed a loss‐of‐function phenotype, as evidenced by high Rh123 fluorescence independent from TQD treatment. These results clearly demonstrate that the wild‐type MDR1 carrier proteins from both dog and cat mediate efficient and specific efflux of Rh123, while the respective mutant proteins both lack measurable transport activity for Rh123 in this efflux assay.

### 3.4 IC_50_ Determination for MDR1 Carrier Inhibitors

**FIGURE 5:**
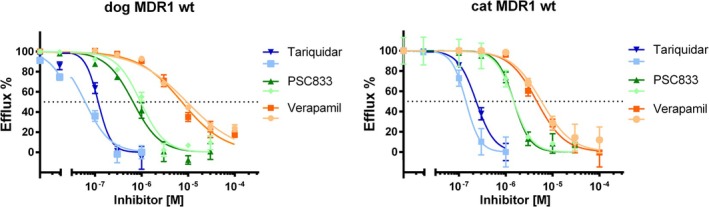
Inhibition of the wild‐type MDR1 carrier proteins of dog and cat with the MDR1 carrier inhibitors verapamil, PSC833, and tariquidar. Inhibition of the Rh123 efflux activity of the wild‐type MDR1 carrier proteins of dog and cat, stably expressed in HEK293 cells after tetracycline treatment, was performed at increasing concentrations of the following MDR1 inhibitors: Verapamil (orange line), valspodar (PSC833) (green line) and tariquidar (blue line). Half‐maximal inhibitory concentrations (IC_50_) were calculated by nonlinear regression (GraphPad Prism). The IC_50_ values were determined in two independent experiments. All data represent means ± SD of quadruplicate determinations. IC_50_ values (μM) for dog MDR1 wt were as follows: Verapamil, 7.3 and 9.4 μM; PSC833, 0.66 and 0.95 μM; tariquidar 0.06 and 0.12 μM. IC_50_ values (μM) for cat MDR1 wt were as follows: Verapamil, 4.6 and 5.8 μM; PSC833, 1.5 and 1.5 μM; tariquidar, 0.14 and 0.23 μM.

To further characterize and compare the functional activity of wild‐type canine and feline MDR1 carrier proteins, Rh123 efflux assays were performed in the presence of increasing concentrations of three established MDR1 carrier inhibitors, and half‐maximal inhibitory concentrations (IC_50_) were determined. Verapamil, a first‐generation MDR1 carrier inhibitor, exhibited relatively weak inhibition of MDR1‐mediated Rh123 efflux, with IC_50_ values of 7.3 and 9.4 μM for the canine MDR1 carrier, and 4.6 and 5.8 μM for the feline MDR1 carrier in two independent experiments (Figure 5). In contrast, the second‐generation inhibitor PSC833 demonstrated higher potency, yielding IC_50_ values of 0.66 and 0.95 μM for the canine MDR1 carrier, and 1.5 and 1.5 μM for the feline MDR1 carrier. The third‐generation MDR1 inhibitor tariquidar (TQD) was the most potent, with the lowest IC_50_ values observed: 0.06 and 0.12 μM for canine MDR1 and 0.14 and 0.23 μM for feline MDR1.

**FIGURE 6:**
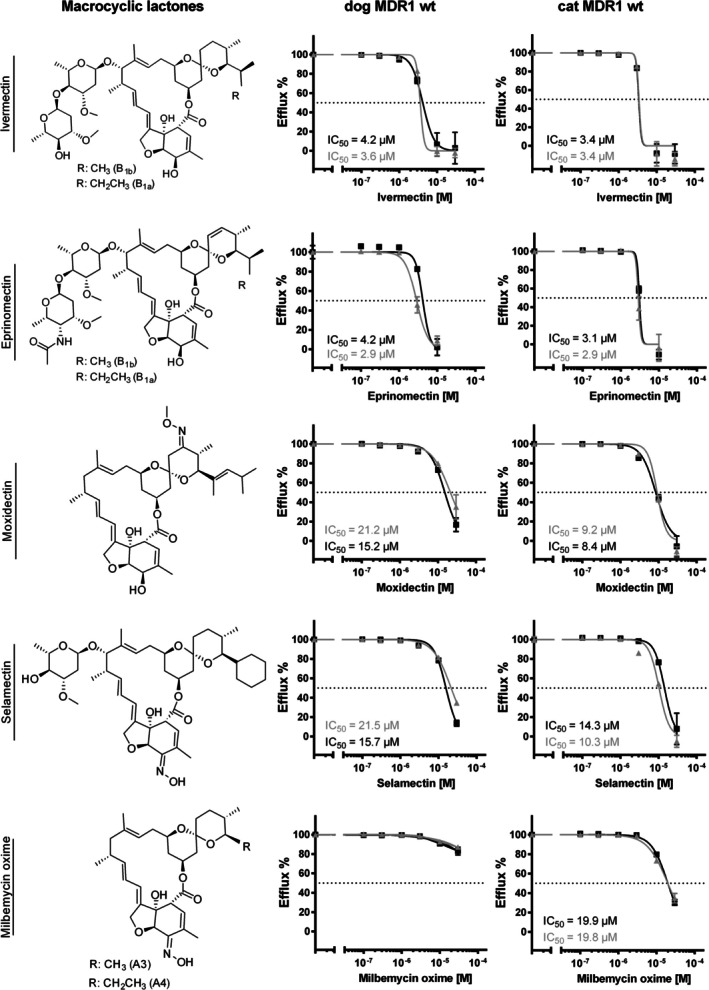
Inhibition of the wild‐type MDR1 carrier proteins of dog and cat with antiparasitic drugs from the macrocyclic lactone class. The wild‐type (wt) MDR1 carrier proteins of dog and cat were expressed in stably transfected HEK293 cells after tetracycline treatment. The efflux of 5 μM Rh123 was analyzed at increasing concentrations of the indicated macrocyclic lactones ivermectin, eprinomectin, moxidectin, selamectin, and milbemycin oxime. The Rh123 efflux in the absence of TQD was set to 100% and the efflux rate in the presence of 1 μM TQD was set to 0%. Determination of the IC_50_ values was done by nonlinear regression (GraphPad Prism). The IC_50_ values were determined in two independent experiments. All data represent means ± SD of quadruplicate determinations.

In addition to these established MDR1 carrier inhibitors, selected antiparasitic drugs from the macrocyclic lactone and isoxazoline classes were analyzed for their interaction with the canine and feline MDR1 carriers. As shown in Figure 6, ivermectin and eprinomectin displayed strong and comparable inhibitory effects on MDR1 carrier activity in both species, with IC_50_ values ranging from 2.9 to 4.2 μM. Moxidectin and selamectin were less potent, with IC_50_ values ranging from 8.4 to 21.5 μM. Notably, while ivermectin and eprinomectin showed no apparent species‐specific differences, selamectin and, to a greater extent, moxidectin exhibited slightly higher potency against the feline MDR1 carrier compared to the canine MDR1 carrier (Figure 6). Interestingly, milbemycin oxime showed measurable inhibition of the feline MDR1 carrier but failed to inhibit the canine MDR1 carrier at concentrations up to 30 μM. Consequently, an IC_50_ value could not be determined for milbemycin oxime at the canine MDR1 carrier protein.

**FIGURE 7:**
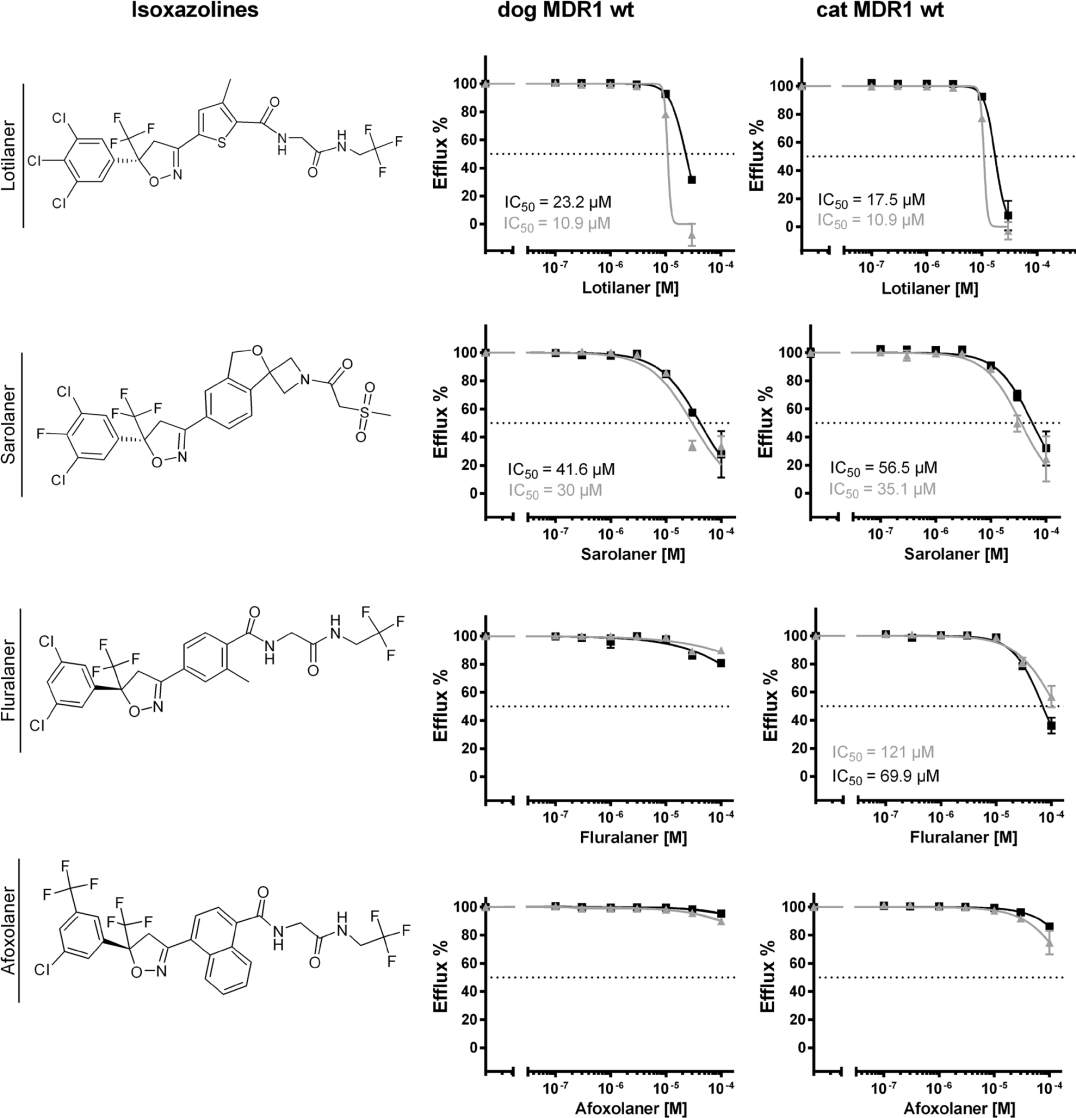
Inhibition of the wild‐type MDR1 carrier proteins of dog and cat with antiparasitic drugs from the isoxazoline class. The wild‐type (wt) MDR1 carrier proteins of dog and cat were expressed in stably transfected HEK293 cells after tetracycline treatment. The efflux of 5 μM Rh123 was analyzed at increasing concentrations of the indicated isoxazoline drugs lotilaner, sarolaner, fluralaner, and afoxolaner. The Rh123 efflux in the absence of TQD was set to 100% and the efflux rate in the presence of 1 μM TQD was set to 0%. Determination of the IC_50_ values was done by nonlinear regression (GraphPad Prism). The IC_50_ values were determined in two independent experiments. All data represent means ± SD of quadruplicate determinations.

Among the isoxazoline compounds, afoxolaner showed no detectable interaction with the MDR1 carriers from either species within the analyzed inhibitor concentration range (Figure 7). Fluralaner exhibited weak inhibition of the feline MDR1 carrier, with IC_50_ values of 69.9 μM and > 100 μM in two independent experiments but did not inhibit the canine MDR1 carrier up to the maximum tested concentration of 100 μM. Sarolaner inhibited both canine and feline MDR1 carriers with no apparent species preference, with IC_50_ values ranging from 30 to 56.5 μM. The most potent MDR1 carrier inhibitor among the tested isoxazoline compounds was lotilaner, with IC_50_ values between 10.9 and 23.2 μM for both feline and canine MDR1 carrier proteins, comparable in potency to selamectin.

### 3.5 Characterization of MDCKII^MDR1ko^ and Stably MDR1‐Transfected MDCKII^MDR1ko^ Cells

**FIGURE 8:**
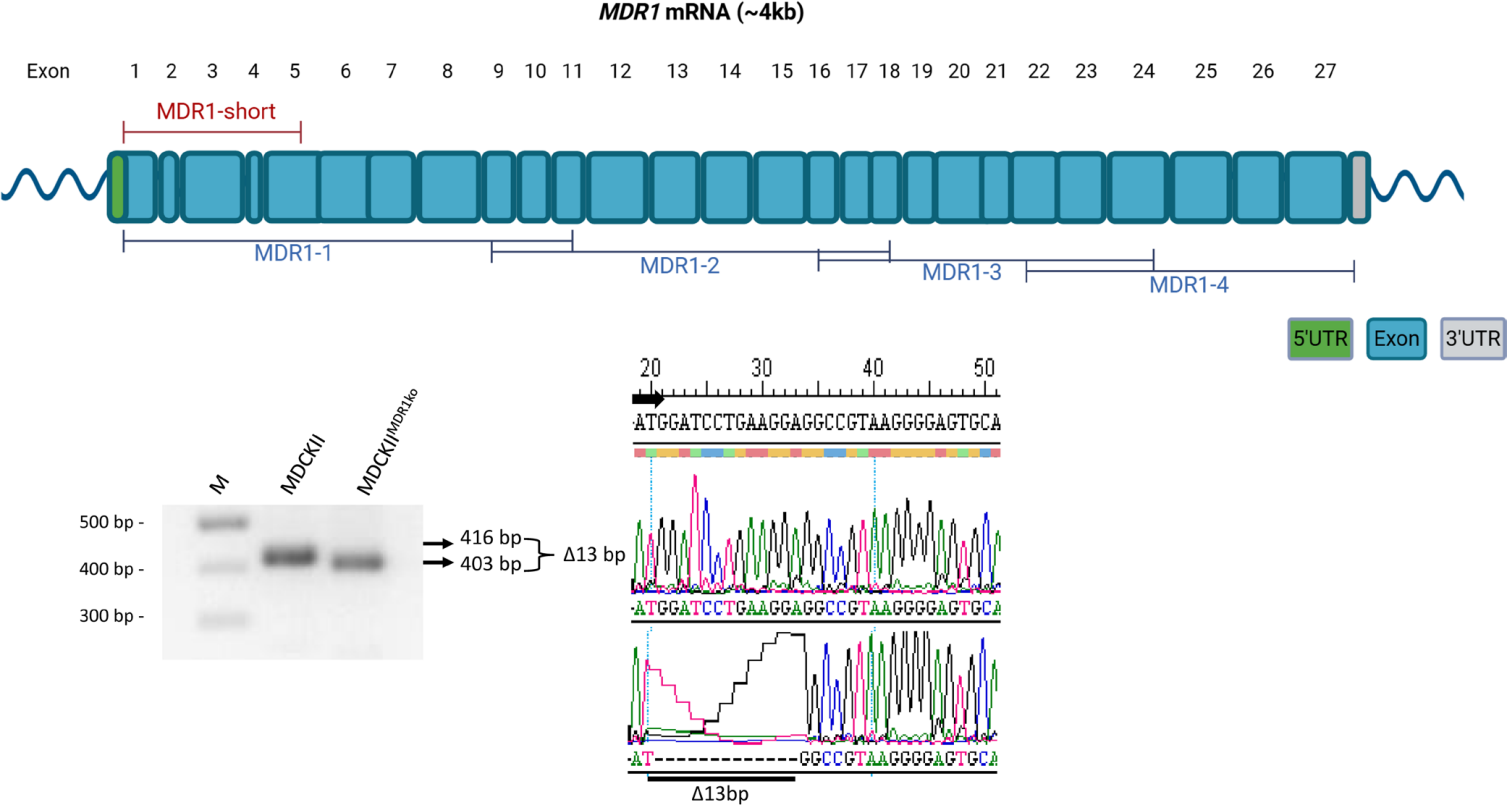
Characterization of the MDCKII^MDR1ko^ cell line. RNA was isolated from MDCKII and MDCKII^MDR1ko^ cells and was used for full‐length PCR amplification and sequencing of the full‐length *MDR1* mRNA transcript with the oligonucleotide primers listed in Table 2 and indicated in the diagram. The oligonucleotide primer pair MDR1‐short is flanking the site of CRISPR/Cas9 mutation in the *MDR1* gene in exon 1. PCR products of 416 bp (MDCKII) and 403 bp (MDCKII^MDR1ko^) were subjected to sequencing. The chromatograms confirmed the CRISPR/Cas9 mutation of 13 bp within the reading frame of MDR1 in exon 1.

**FIGURE 9:**
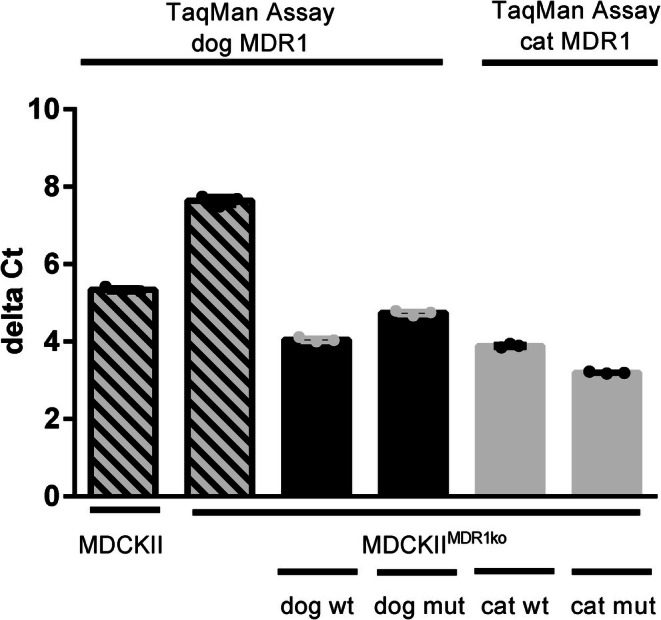
*MDR1* mRNA expression in the transfected MDCKII^MDR1ko^ cell lines. The *MDR1* mRNA expression was quantified by real‐time PCR in the MDCKII^MDR1ko^ cells stably transfected with the MDR1 wild‐type and mutant constructs of dog and cat, in the non‐transfected MDCKII^MDR1ko^ cells and in the parent MDCKII cells. The species‐specific TaqMan probes for real‐time PCR are listed in Table 1. The *MDR1* mRNA expression data were analyzed by the ΔCt method with *B2M* as the common housekeeper.

In addition to the HEK293 cell model, MDCKII cells can be used for MDR1 transport studies. The parental MDCKII cell line is of canine origin and endogenously expresses the canine MDR1 carrier protein at appreciable levels, which limits its suitability for analyzing recombinant MDR1 carrier proteins from other species after transfection. However, MDCKII cells offer a significant advantage in that they form tight monolayers, making them ideal for transcellular transport studies using the transwell systems. To overcome the limitation of endogenous MDR1 expression while leveraging the transcellular transport capability, we used a commercially available MDCKII^MDR1ko^ cell line in which the canine *MDR1* gene was inactivated via CRISPR/Cas9‐mediated gene editing. As a first step, we aimed to localize and characterize the induced mutation. This was achieved through PCR amplification of the full‐length canine *MDR1* coding sequence using four primer pairs covering the entire open reading frame (see Table 2). The resulting PCR products were sequenced and compared to the wild‐type *MDR1* sequence. Sequence analysis revealed a 13‐base‐pair deletion in exon 1, resulting in a frameshift and the introduction of a premature stop codon. This mutation effectively prevents expression of the endogenous canine MDR1 transporter in MDCKII^MDR1ko^ cells. As illustrated in Figure 8, the deletion was detected by a band shift following PCR amplification with the primer pair MDR1‐short. Subsequent sequencing confirmed a 13 bp excision directly upstream of the sequence 5′‐GGCCGTAAGGGGAGTGCA‐3′ in exon 1 of the *MDR1* gene. This knockout cell line was then used for stable integration of a Flp recombinase target (FRT) site. Using Flp‐mediated recombination, expression constructs encoding wild‐type and mutant MDR1 carrier proteins from dog and cat were stably integrated into the genome of the MDCKII^MDR1ko^‐FRT cells. The resulting transfected MDCKII^MDR1ko^ cell lines constitutively expressed the wild‐type canine *MDR1* mRNA, the mutant canine *MDR1* mRNA (nt230(del4)), the wild‐type feline *MDR1* mRNA, and the mutant feline *MDR1* mRNA (nt1930(del2)) transcripts, analogous to the stably transfected HEK293 cell lines described above. Expression of the species‐specific *MDR1* mRNA transcripts was analyzed by real‐time PCR using TaqMan gene expression assays: assay cf02693309_m1 for canine *MDR1*, assay MDR1CatbpExon15 for feline *MDR1*, and assay cf02659079_m1 for canine *B2M* as a reference gene to calculate ΔCt values. As shown in Figure 9, low‐level expression of the truncated endogenous *MDR1* mRNA (containing the 13 bp deletion) was still detectable in MDCKII^MDR1ko^ cells. In contrast, the recombinant wild‐type and mutant canine and feline *MDR1* mRNA transcripts were strongly expressed, with ΔCt values around 4, indicating high transcription levels. For feline MDR1, the mutant *MDR1* mRNA transcript construct was expressed more robustly than the wild‐type *MDR1* mRNA transcript.

**TABLE 2:** Primers used for full‐length sequencing of the *MDR1* mRNA transcript from MDCKII^MDR1ko^ cells.

Primer pair ID	Forward primer (5′ → 3′)	Reverse primer (5′ → 3′)	Amplicon (bp)
MDR1‐1	AAG GAA AGC CCG AGG TGA CGA TG	CTG TCT GCC CAC TCT GAA CCT TC	1293
MDR1‐2	CGC AAG AGG AGC AGC TTA TGA AAT C	GGA TCT CCC CAG CTT TGC CAA ATG	1280
MDR1‐3	GGA GGA TTC TGA AGC TGA ACT CAA C	GGA TGT CTG GTC GAG TGG GAT AG	1059
MDR1‐4	CAC ACA TCT TCG GGG TCT CAT TTT C	CGG CCA CAG TTC ACT AGC GTT TTG	1065
MDR1‐short	AAG GAA AGC CCG AGG TGA CGA TG	GTA AGC AGC CAC CAG CAC GC	416

### 3.6 Transwell Transport Experiments in Stably Transfected MDCKII^MDR1ko^ Cells

**FIGURE 10:**
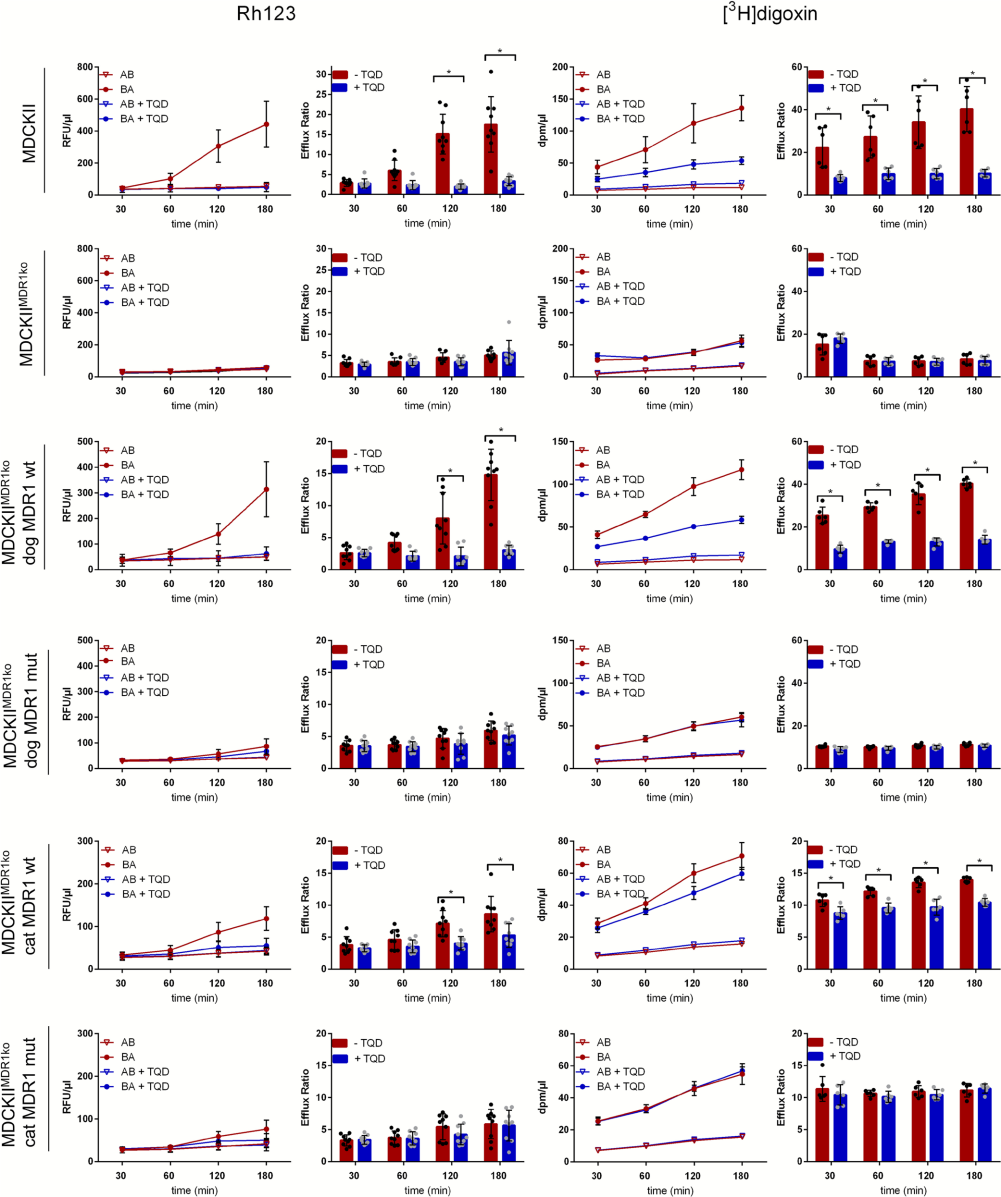
Transwell transport experiments with Rh123 and [^3^H]digoxin in MDCKII cells. MDCKII cells lacking endogenous canine MDR1 (MDCKII^MDR1ko^) were stably transfected with the canine MDR1 wild‐type (dog MDR1 wt), canine MDR1 knockout (dog MDR1 mut), feline MDR1 wild‐type (cat MDR1 wt), and feline MDR1 knockout (cat MDR1 mut) constructs, respectively. Non‐transfected MDCKII^MDR1ko^ cells and the parent MDCKII cell lines were additionally analyzed for control. Transport experiments were performed in both apical‐basolateral (AB) and basolateral‐apical (BA) directions in the presence (blue bars) and absence (red bars) of the MDR1 inhibitor tariquidar (TQD). Samples from the donor chamber and the receiver chamber were taken at different time points. Fluorescence of Rh123 was quantified using a fluorescence plate reader. The amount of radiolabeled [^3^H]digoxin in the samples was measured by liquid scintillation counting. Efflux ratios (ER) were calculated as described in the Material and Methods section. Data are means ± SD from three independent experiments each with triplicate determinations (*n* = 9, Rh123) or from two independent experiments each with triplicate determinations (*n* = 6, [^3^H]digoxin). *Significantly higher in the absence of TQD compared to the presence of TQD according to two‐way ANOVA with *p* < 0.05.

The parent MDCKII and the stably transfected MDCKII^MDR1ko^ cell lines were then used for experiments in the transwell system. The transwell transport experiments with Rh123 (Figure 10, left panel) and [^3^H]digoxin (Figure 10, right panel) as the probe substrates clearly demonstrated time‐dependent intact transport function for the parent MDCKII cells as well as for the MDCKII^MDR1ko^ cells, stably overexpressing the wild‐type *MDR1* mRNAs from dog (dog MDR1 wt) and cat (cat MDR1 wt). This was evidenced by a higher apparent permeability coefficient (*P*_app_) in the basolateral to apical direction compared to the apical to basolateral direction, resulting in significantly higher efflux ratios (ER) in the absence of the MDR1 inhibitor TQD. In contrast, TQD completely abolished transcellular transport of Rh123 and [^3^H]digoxin in all three cell lines as expected. In the case of the non‐transfected MDCKII^MDR1ko^ cells and the MDCKII^MDR1ko^ cells overexpressing the mutant *MDR1* mRNAs from dog (dog MDR1 mut) and cat (cat MDR1 mut), comparable ER values were determined in the presence and absence of TQD, without any significant difference (Figure 10).

### 3.7 MDR1 Genotyping in 800 Cats From Germany

Because the prevalence of the *MDR1* mutation in cats has been less well analyzed than in dogs, we conducted an *MDR1* genotyping study on 800 cats from Germany covering 28 different cat breeds. Among them, the *MDR1* nt1930(del2) (syn. ABCB1 1930_1931del TC) mutation was detected in four purebred Maine Coon cats and in one European shorthair cat. All five animals revealed the heterozygous (MDR1+/−) genotype. Cats homozygous for this mutation (MDR1−/−) were not identified. Thus, occurrence of the mutant *MDR1* nt1930(del2) allele was at 0.625% in the present study (Table 3).

**TABLE 3:** MDR1 genotyping in 800 cats of different breeds from the patient collective of a university veterinary clinic in Germany. The following nt1930(del2) genotypes are listed: (+/+), homozygous wild‐type; (+/−), heterozygous mutation; (−/−), homozygous mutation.

Cat breed	*MDR1* nt1930(del2) genotype, absolute numbers		
	(+/+)	(+/−)	(−/−)
Bengal	25	—	—
Birman	11	—	—
Brazilian shorthair	2	—	—
British shorthair	98	—	—
British longhair	3	—	—
Burmese	2	—	—
Chartreux	1	—	—
Carthusian	3	—	—
European shorthair	447	1	—
European longhair	2	—	—
Exotic shorthair	1	—	—
German Rex	1	—	—
Maine Coon	80	4	—
Norwegian Forest Cat	14	—	—
Oriental shorthair	2	—	—
Others^a^	48	—	—
Persian	10	—	—
Ragdoll	8	—	—
Russian Blue	4	—	—
Savannah	1	—	—
Scottish Fold	8	—	—
Siamese	9	—	—
Siberian Forest Cat	4	—	—
Singapura	1	—	—
Somali	1	—	—
Sphinx	2	—	—
Thai	1	—	—
Turkish Angora	5	—	—
Ural Rex	1	—	—
Total	795	5	0

^a^ Others include cats listed as mix or unknown breed.

## 4 Discussion

In this study, the influence of the nt230(del4) *MDR1* gene mutation in dogs and the nt1930(del2) *MDR1* gene mutation in cats on the transport function of the MDR1 carrier proteins was investigated in cell culture models. In particular, the question of whether the previously described 2‐bp deletion mutation in the *MDR1* gene of cats leads to a complete loss of function of the MDR1 carrier protein has not yet been definitively answered. Although the mutation results in a frameshift that truncates the MDR1 carrier protein to about half of its normal size, it has not been entirely ruled out that two mutated cat MDR1 carrier proteins could form an artificial homodimer with residual activity, as has been observed in other members of the ABC transporter family, such as ABCG2 (Xu et al. 2004). To determine the influence of nt230(del4) *MDR1* gene mutation in dogs and the nt1930(del2) *MDR1* gene mutation in cats on the MDR1 carrier efflux function, in vitro transport experiments were conducted on stably transfected HEK293 and MDCKII cells. While HEK293 is a well‐established cell model for in vitro transport studies (Geyer, Döring, et al. 2007), MDCKII were additionally used for analyzing transcellular transport, due to their ability to form epithelial monolayers with functional tight junctions when cultured on membrane filters in the transwell system (Ye et al. 2020). As the interpretation and analysis of data in many cell lines is complicated by the presence of endogenous transporters such as endogenous canine *MDR1* mRNA and MDR1 carrier expression in MDCKII cells, we additionally used *MDR1* mutant MDCKII cells with absent MDR1 carrier expression (MDCKII^MDR1ko^) (Karlgren et al. 2017). Using the transwell transport assays, we showed complete absence of transcellular Rh123 or [^3^H]digoxin transport indicating that the *MDR1* gene mutation in the MDCKII^MDR1ko^ cells results in a complete loss of MDR1 carrier activity, whereas this activity is retained in the parental MDCKII cells. Finally, expression of the mutant MDR1 constructs of dog and cat in HEK293 and MDCKII^MDR1ko^ cells clearly demonstrated a complete loss of function phenotype of the mutant MDR1 carrier proteins (Figures 4 and 10). Furthermore, the established stably dog and cat MDR1‐transfected MDCKII^MDR1ko^ cells can be used to identify additional canine and feline MDR1 carrier substrates in subsequent studies. A comparable approach was previously reported by Mealey et al. (2017), who established a cell line for assessing drugs as canine MDR1 carrier substrates (Mealey et al. 2017). It is acknowledged that there are both similarities and differences in the MDR1 substrate recognition pattern between distinct species (Zolnerciks et al. 2011). This also became evident for the different inhibition pattern for milbemycin oxime and fluralaner for the MDR1 carriers of cat and dog in the present study (Figures 6 and 7). Consequently, it is not possible to assume that MDR1 carrier substrates identified in humans or rodents are automatically substrates of the canine or feline MDR1 carrier proteins. Further research is required to identify additional substrates of the canine and feline MDR1 carriers, to avoid adverse drug reactions in animals with *MDR1* gene mutation when treated with drugs transported by the MDR1 carrier proteins.

MDR1‐transfected HEK293 cells were used as a well‐established cell model for transport studies. These cells over‐expressing the wild‐type MDR1 carrier proteins of dog and cat are suitable to investigate drug interactions at the level of MDR1 transport. In the present study, we used this model to analyze drug binding to the MDR1 carrier proteins of dog and cat for antiparasitic drugs of the macrocyclic lactone and isoxazoline classes. In this part of the study, we showed that in addition to ivermectin, other structurally similar compounds from the macrocyclic lactone class also interact with the MDR1 carrier proteins of dog and cat. It was demonstrated that ivermectin and eprinomectin had the lowest IC_50_ value and were, therefore, the best inhibitors of Rh123 transport, whereas moxidectin, selamectin, and milbemycin oxime were poorer inhibitors of the MDR1 carrier proteins of dog and cat (Figure 6). Macrocyclic lactones can be divided into two large groups, the avermectins (ivermectin, eprinomectin, selamectin) and the milbemycins (moxidectin, milbemycin oxime), and have a macrocyclic lactone ring as a common backbone. In contrast to the milbemycins, the avermectins possess additional sugar groups at C_13_ on the macrocyclic ring, while the milbemycins represent aglycones (Prichard et al. 2012). A comparison of the structures of ivermectin and eprinomectin (two sugar substitutions on the macromolecule) and of moxidectin and milbemycin oxime (no sugar substitution on the macromolecule) (Figure 6) with the measured IC_50_ values in the present study led to the conclusion that the sugar substitutions might have an influence on the affinity of the substances to the MDR1 carrier proteins of dog and cat. In consideration of its chemical structure, selamectin (one sugar substitution) might have an intermediate position. It is well established that ivermectin is not only an MDR1 carrier substrate but also an inhibitor of the MDR1 carrier protein (Didier and Loor 1996). Even more, in human MDR1‐overexpressing vincristine‐resistant Caco‐2 cells (Caco‐2VCR), ivermectin was shown to be one of the most potent inhibitors of the MDR1 carrier (Eneroth et al. 2001). In contrast, moxidectin was found to be a poor inhibitor of MDR1‐mediated Rh123 transport in Caco‐2 cells and canine peripheral blood lymphocytes (Griffin et al. 2005). Furthermore, the effect of various macrocyclic lactones on the ATPase activity of the MDR1 carrier, stimulated by verapamil in membrane vesicles from DC‐3F‐/ADX MDR1‐overexpressing cells, was analyzed (Lespine et al. 2007). In these experiments, ivermectin showed significantly higher affinity to the MDR1 carrier than selamectin with *K*_i_ value of 0.05 μM for ivermectin and of 1.0 μM for selamectin. The authors concluded that the size of the sugar moiety on the macromolecule (two sugar substitutions for ivermectin, one sugar substitution for selamectin) could be responsible for these different affinities. These data from previous in vitro studies support the results from the current study. It is notable that the IC_50_ value of selamectin is higher in comparison to both ivermectin and eprinomectin, and it is comparable to moxidectin. Furthermore, in vivo studies have demonstrated that the concentration ratios in the brain (*mdr1* knockout vs. wild‐type mice) are significantly less pronounced with selamectin (5‐ to 10‐fold) than the corresponding ratios with ivermectin (36‐ to 60‐fold), also supporting higher affinity transport via the murine MDR1 carrier for ivermectin than for selamectin (Geyer et al. 2009). However, other studies have demonstrated that selamectin is an equally potent MDR1 carrier substrate/inhibitor as ivermectin (Griffin et al. 2005; Brayden and Griffin 2008). In addition, it must be mentioned that selamectin is tolerated in ivermectin‐sensitive Collies at higher doses than ivermectin (Bishop et al. 2000; Novotny et al. 2000). The administration of ivermectin at doses > 120 μg/kg to ivermectin‐sensitive Collies resulted in the onset of neurological toxicity. In contrast, oral doses of selamectin exceeding 15 mg/kg remained without clinical symptoms of neurological toxicity (Geyer and Janko 2012).

Compared with ivermectin, antiparasitic drugs of the isoxazoline class appear to have lower affinities toward the MDR1 carrier proteins of dog and cat in the present study (Figure 7). Due to solubility restrictions, the highest inhibitory concentration of fluralaner and afoxolaner was at 100 μM. At this concentration, no inhibition of the MDR1 carrier was observed for afoxolaner. Fluralaner showed inhibition only for the feline MDR1 carrier with an IC_50_ value of 69.9 μM and > 100 μM. In addition, in vivo studies have demonstrated that the oral administration of afoxolaner, either as a monotherapy or in combination with milbemycin oxime, is safe for dogs with homozygous *MDR1* gene mutation (Drag et al. 2022). A similar study was conducted with oral administration of fluralaner in Collies with homozygous *MDR1* gene mutation and confirmed treatment safety without neurological toxicity (Walther et al. 2014).

To determine the prevalence and breed distribution of the *MDR1* gene mutation in cats from Germany, 800 cats from the patient collective of the Clinic for Small Animals of the Justus Liebig University Giessen were examined. The study revealed the presence of the heterozygous *MDR1* gene mutation (MDR1+/−) in one European Shorthair cat and four Main Coon cats. These results were expected, as previous studies have already demonstrated the presence of the *MDR1* gene mutation in these two cat breeds (Mealey et al. 2021; Anderson et al. 2022; Nürnberger et al. 2022). As our data only refers to cats from Germany and the two other studies analyzed the *MDR1* gene mutation in the cat population from the United States and some other countries, we aimed to directly compare this data in the additional Table 4. In the study by Mealey et al. (2021), 1006 cats were *MDR1* genotyped and a heterozygous *MDR1* nt1930(del2) (ABCB1 1930_1931del TC) gene mutation was detected in 47 animals. In this study, Domestic Shorthair cats were most affected, but the mutation was not found in any of the 20 Main coon cats analyzed. The second study by Anderson et al. (2022) analyzed 11,036 cats from the United States (54.9% of the cats), Finland (17.4%), Canada (5.3%), the United Kingdom (3.5%), Norway (3.5%), Sweden (3.3%), Russia (2.5%), and France (1%). The *MDR1* nt1930(del2) (ABCB1 1930_1931del TC) mutation was detected in 123 cats with the heterozygous (MDR1+/−) genotype and in 3 cats with the homozygous *MDR1* mutation (MDR1−/−). In this study, the mutation was also quite frequently found in Maine Coon cats (Maine Coon breed 107/1971 and the Main Coon Polydactyl breed 10/150). The fact that only three cats were genotyped homozygous for the *MDR1* mutation in this large study suggests that cats with homozygous *MDR1* mutation seem to be extremely rare (Anderson et al. 2022). However, if such cats are treated with ivermectin or eprinomectin, severe neurological toxicity can occur (Mealey et al. 2021, 2024, 2025; Nürnberger et al. 2022). Neurological toxicity has been reported only in cats with homozygous *MDR1* mutation that received either subcutaneous ivermectin (Mealey and Burke 2015) or topical eprinomectin (Mealey et al. 2021, 2024, 2025). At present, there are approved drugs for ectoparasites and endoparasites in cats available in the EU that contain eprinomectin. Although numerous substances like moxidectin, milbemycin oxime, emodepside, cyclosporine A, butorphanol, vincristine and doxorubicin are known to cause clinical signs of neurological toxicity in dogs with the *MDR1* gene mutation (Mealey et al. 2001, 2017, 2023; Geyer, Döring, Godoy, Moritz, et al. 2005; Geyer, Klintzsch, et al. 2007; Gaens et al. 2019), no additional critical drugs other than ivermectin and eprinomectin have been identified in cats with *MDR1* gene mutation so far. However, it cannot be ruled out that drugs provoking adverse drug reactions in *MDR1* mutant dogs may also be less tolerated by cats with *MDR1* gene mutation. Consequently, it is important for veterinarians to be aware of this genetic *MDR1* mutation, even though it is rare in the cat population.

**TABLE 4:** Worldwide frequency of the nt1930(del2) *MDR1* mutation in cats from selected breeds. Overview of the prevalence of the nt1930(del2) *MDR1* mutation in cats as reported by studies worldwide. The following nt1930(del2) genotypes are listed: (+/+), homozygous wild‐type; (+/−), heterozygous mutation; (−/−), homozygous mutation.

Study	Table 1 from Mealey et al. (2021)^a^			Table 2 from Anderson et al. (2022)^b^			Table 3 from the present study^c^		
	*MDR1* nt1930(del2) genotype, absolute numbers								
Cat breed	(+/+)	(+/−)	(−/−)	(+/+)	(+/−)	(−/−)	(+/+)	(+/−)	(−/−)
Balinese	—	—	—	74	2	0	—	—	—
Domestic shorthair	581	30	0	—	—	—	—	—	—
Domestic medium hair	80	5	0	—	—	—	—	—	—
Domestic longhair	125	7	0	—	—	—	—	—	—
European shorthair	—	—	—	—	—	—	447	1	0
European longhair	—	—	—	—	—	—	2	0	0
Main Coon	20	0	0	1864	104	3	80	4	0
Main Coon Polydactyl	—	—	—	140	10	0	—	—	—
Others	21	2	0	612	5	0	48	0	0
Ragdoll	9	1	0	1115	0	0	8	0	0
Russian Blue	7	1	0	64	0	0	4	0	0
Siamese	26	1	0	145	1	0	9	0	0
Turkish Angora	—	—	—	109	1	0	5	0	0

*Note:* Others, include cats listed as mix or unknown breed; numbers represent total amount. Countries from which the tested cats originate: ^a^USA (100%); ^b^United States of America (54.9%), Finland (17.4%), Canada (5.3%), United Kingdom (3.5%), Norway (3.5%), Sweden (3.3%), Russia (2.5%), France (1%); ^c^Germany (100%).

Abbreviation: —, no data available.

As an outlook, the cell lines established in the present study, together with the generated IC_50_ data, demonstrate that our in vitro systems reliably detect interactions between diverse active substances and the canine or feline MDR1 carrier proteins. Our cell models enable the early identification of compounds with a risk of MDR1‐mediated drug–drug interactions or with relevant efflux at the blood–brain barrier. This, in turn, allows problematic drug candidates to be optimized or excluded during pre‐clinical development prior to in vivo testing. Consequently, the system provides a robust basis for evaluating the safety profiles of veterinary therapeutics and supports efforts to reduce the need for neurotoxicity studies in animals.

## Author Contributions

M.H. and J.G. conceived and designed the project; L.S. performed all in vitro cell culture experiments; S.O. performed the proteomics analysis; L.S., D.N., and M.B. performed the MDR1 genotyping; A.M. provided the blood samples for MDR1 genotyping; L.S., S.O., and J.G. analyzed and interpreted the data. L.S. and J.G. wrote the manuscript; L.S. prepared the figures; M.H. and J.G. critically edited and revised the manuscript. All authors contributed to the article and approved the final version.

## Funding

Funding for this study was received from the Bundesamt für Verbraucherschutz und Lebensmittelsicherheit (BVL, Braunschweig/Berlin, Germany) via the pharmacovigilance program. The mass spectrometric‐based proteomic analysis was funded by the German Research Foundation (project number: 505943254).

## Ethics Statement

The authors confirm that the ethical policies of the journal, as noted on the journal's author guidelines page, have been adhered to and the appropriate ethical review committee approval has been received. The authors confirm that they have adhered to European standards for the protection of animals used for scientific purposes. The use of surplus diagnostic blood samples for MDR1 analysis and genotyping was reviewed and approved by the local animal welfare authority (Regierungspräsidium Giessen) with the registration number: 19 c 20 15 h 02 Gi 18/11 kTV 10/2021.

## Conflicts of Interest

MDR1 genotyping of dogs and cats is commercially available from the TransMIT GmbH center for Pharmacogenetic Diagnostics PGvet (scientific director Prof. Dr. Joachim Geyer) at the Institute of Pharmacology and Toxicology, Justus Liebig University of Giessen.

## Data Availability

All data and original contributions of the presented study are included in the article; further inquiries can be directed at the corresponding author.
